# Nonalcoholic Fatty Liver Disease: Identifying the Disease Burden in Sri Lanka

**DOI:** 10.5005/jp-journals-10018-1263

**Published:** 2018-05-01

**Authors:** Anuradha S Dassanayake

**Affiliations:** Faculty of Medicine, University of Kelaniya, Kelaniya, Sri Lanka

**Keywords:** Nonalcoholic fatty liver disease, Prevalence, Sri Lanka.

## Abstract

Nonalcoholic fatty liver disease (NAFLD) is becoming one of the most important causes for chronic liver disease and also hepatocellular carcinoma (HCC) in Sri Lanka. This tendency is also recognized worldwide. More than half of the middle-aged and elderly adults in urban Sri Lanka have ultrasonic evidence of NAFLD. The NAFLD is also identified in population from rural areas of Sri Lanka and also in children. Nonalcoholic steatohepatitis (NASH) cirrhosis is the most common cause of referral for liver transplantation in Sri Lankans. The NASH is also the most common cause for rejecting potential donors for liver transplantation in Sri Lanka. Patients who underwent liver transplantation for cryptogenic cirrhosis developed evidence of NASH following liver transplantation. Recent evidence suggests that there is a genetic component to NAFLD. PNPLA3, a single gene polymorphism linked to the short arm of chromosome 22, is associated with the severity of NAFLD. The presence of this genetic polymorphism appears to carry higher risk of patients with NAFLD developing NASH with fibrosis cirrhosis and hepatocellular carcinoma. In a large population-based study from Sri Lanka, there was a tendency to develop NAFLD associated with this genetic polymorphism. In a population-based study, NAFLD was identified as an independent risk factor for development of diabetes. This association is recognized worldwide now. Most patients with HHC in Sri Lanka developed it on a back ground of cryptogenic cirrhosis. At the same time, the prevalence of the markers for hepatitis B and C was rare in Sri Lankan patients with HCC.

**How to cite this article:** Dassanayake AS. Nonalcoholic Fatty Liver Disease: Identifying the Disease Burden in Sri Lanka. Euroasian J Hepato-Gastroenterol 2018;8(1):69-72.

## INTRODUCTION

Nonalcoholic fatty liver disease is one of the most important health-related issues in Sri Lanka. Available evidence suggests that NAFLD is the most common chronic liver diseases in the country. In a large hospital-based study carried out recently, cryptogenic cirrhosis and alcoholic liver disease were found to be the two predominant etiological factors for cirrhosis in Sri Lanka.^1^ Many consider NASH, the severe form of NAFLD with hepatic inflammation, to be the predominant etiology for cryptogenic cirrhosis. Currently, NASH is believed to be responsible for the most number of patients with cryptogenic cirrhosis worldwide. In a more recent study, cryptogenic cirrhosis was the predominant cause for listing for liver transplantation in Sri Lanka.^2^ Cryptogenic cirrhosis is also considered to be the most common etiology responsible for HCC in Sri Lanka.^3^

Nonalcoholic fatty liver disease represents a spectrum of liver disease encompassing simple fatty infiltration of the liver parenchyma (steatosis), fatty infiltration and inflammation NASH, and cirrhosis, in the absence of excessive alcohol consumption.^4,5^ Nonalcoholic fatty liver disease is now considered to be the most common chronic liver disease in the world.^6^ The worldwide prevalence of NAFLD varies between 15 and 40% in different populations. However, since the diagnosis of NASH requires histological assessment, the prevalence of NASH is unclear and is estimated to be around 10 to 30% of NAFLD patients. A percentage of patients with NASH will end up with end-stage liver disease and HCC. It is now recognized that NAFLD is the most common cause of liver dysfunction in developed countries. Nonalcoholic fatty liver disease is on the increase in the developing countries as well. This is most likely due to improving living standards, increasing prevalence of unhealthy eating habits, and sedentary lifestyles. Nonalcoholic fatty liver disease is now considered to be the hepatic component of the metabolic syndrome. Risk factors for NAFLD such as diabetes mellitus and obesity are rapidly on the rise worldwide, resulting in the increasing prevalence of NAFLD.

In South Asia, traditionally the chronic liver disease burden has been due to hepatitis B and C virus. Surprisingly, this trend has not been identified from Sri Lanka where the burden of chronic viral hepatitis has always been low. Many factors would have contributed to this tendency. These include the centrally controlled blood banks by the state sector, early introduction of the use of disposable needles to the country, and the relatively low popularity of the intravenous drug abuse in the country. Low popularity of the use of intramuscular route of injections among general practitioners and the public would also have contributed to this trend. The established trend of viral hepatitis in South Asia as the predominant form of chronic liver disease is expected to reverse toward metabolic liver disease, predominantly toward NAFLD in the near future. This is mainly to due to advances in the field of therapeutics in hepatology. This trend is already visible in other South Asian countries like Nepal and India. Improving healthcare facilities would also have contributed immensely to this change. With the advances in the field such as the universal immunization against hepatitis B, and the availability of the cheaper and effective antiviral for hepatitis C, the prevalence of chronic viral hepatitis is expected to go down further gradually, and NASH is expected to become the dominant chronic liver disease in the region in the future.

The prevalence of NAFLD among adults in Sri Lanka is high. In a large population-based study from urban Sri Lanka, among 2,985 adults (aged 35-64 years), 974 (32.6%) were found to have NAFLD on ultrasonography.^7^ The presence of NAFLD was strongly associated with the constituents of the metabolic syndrome. In a similar study, performed in a rural tea-cultivating, physically active, low-income population of adults, the prevalence rate was predictably lower than the urban figures but still significant at 18%.^8^ The urban cohort, when followed up for 7 years and subjected to ultrasonography of the liver again, showed their prevalence of NAFLD had increased dramatically to nearly 66% in this now aging (42-71 years) population.^9^ The annual incidence of NAFLD in this population was 6.6%.

Nonalcoholic fatty liver disease was the most common cause among patients undergoing liver biopsies for unexplained elevation of liver enzymes in a hospital-based study from Sri Lanka. Of the liver biopsies performed on 296 patients with unexplained elevation of liver enzymes, 100 (35.1%) had NASH.^10^ Although traditionally NASH is considered a disease of obese females, there were significant proportion of lean males with NASH in this group. The clinical biochemical and histological features of NASH in this group were similar to features of NASH that were reported in patients from the Western countries. Nonalcoholic fatty liver disease is a significant issue among children in Sri Lanka and worldwide. Advanced hepatic fibrosis and cirrhosis have been reported in Sri Lankan children with NASH.^11^

Recent evidence also suggests that NAFLD is a multisystem disease.^12^ Nonalcoholic fatty liver disease patients carry a higher risk for developing diabetes mellitus, ischemic heart disease, and chronic kidney disease. Majority of patients with NAFLD die of cardiovascular causes. Nonalcoholic fatty liver disease increases the risk of diabetes approximately twofold. In a large population-based study from Sri Lanka, patients with ultrasonically diagnosed NAFLD when followed up for 3 years had higher risk of developing diabetes compared with patients without NAFLD.^13^ Here NAFLD was an independent risk factor for the development of diabetes mellitus. This is considered to be due to hepatic lipid accumulation leading to insulin resistance. It appears as if more severe the NAFLD, the greater the risk of developing diabetes.

Energy excess alone cannot explain NAFLD and NASH always. It is well established that some patients develop these disease entities without demonstrable risk factors like diabetes or obesity, either central or abdominal. Familial clustering of NAFLD is very well recognized. Recently, possible predisposition to fat accumulation in the liver and the progression of liver disease have been demonstrated in individuals with certain genetic polymorphisms. Patients carrying these genes are shown to have severe forms of the illness more often. This predisposition is demonstrated throughout the entire spectrum of NAFLD, from simple steatosis to NASH, fibrosis, cirrhosis, and HCC. This predisposition for NAFLD appears to be expressed even in the absence of the traditional risk factors for NAFLD like obesity and diabetes. Patatin-like phospholipase domain-containing protein 3 (PNPLA3) is one of the genetic polymorphisms most widely studied. There is very robust evidence for this genetic polymorphism for its association with NASH cirrhosis since it was first described in 2008.^14^ Some researchers consider this PNPLA3-associated NASH to be a distinct entity from the common NAFLD that we encounter in patients with multiple metabolic risk factors.^15^ This genetic polymorphism has been described in patients with NAFLD worldwide including South Asia. In a large population-based study from Sri Lanka, a significant association was observed for PNPLA3 polymorphism and susceptibility to NAFLD.^16^ This gene is localized in the long arm of chromosome 22. The genetic defect results in the loss of enzyme activity of triacyl glycerol lipase, resulting in the accumulation of fat in adipocytes and also in hepatocytes. Many other genes that increase the susceptibility to NAFLD have been described since then.

As mentioned earlier, cryptogenic cirrhosis is the predominant etiology for cirrhosis in patients listing for liver transplantation in Sri Lanka. In patients not receiving a liver transplantation, the survival rates among cryptogenic cirrhosis patients and alcoholic cirrhosis patients are similar.^1^ Living donor liver transplantation has become an effective alternative to cadaveric organ shortage in Asia. Preliminary experience from Sri Lanka shows that NAFLD is one of the main reasons for rejecting potential donors of liver.^17^ This is experienced at other liver transplant centers from the region as well. Nonalcoholic steatohepatitis is well recognized to recur in patients who undergo liver transplantation for cryptogenic cirrhosis. In five patients transplanted for cryptogenic cirrhosis, all had evidence of recurrence of varying stages of NAFLD on biopsy during follow-up.^18^

Although hepatitis B and C are the leading causes for HCC worldwide, HCC risk is also higher in other forms of cirrhosis like alcoholic cirrhosis and hemochromatosis. The exact reason for higher risk for HCC in alcoholic cirrhosis is not very well established. It is well recognized that NASH cirrhosis also carries a higher risk of HCC. It is also well recognized that HCC could complicate NASH with fibrosis even in the absence of cirrhosis. Nonalcoholic steatohepatitis cirrhosis appears to be the predominant etiology for HCC in Sri Lanka. In a hospital-based study from Sri Lanka, of the 150 patients with HCC, 61 had alcohol-related cirrhosis and 89 had cryptogenic cirrhosis. None of the patients with HCC were positive for hepatitis B s antigen or hepatitis C antibody.^3^ However, in a previous study, 16% of patients with HCC showed evidence of the presence of hepatitis B and C when investigated for the evidence of molecular markers for hepatitis B deoxyribonucleic acid and hepatitis C ribonucleic acid respectively.^19^ Data from our study suggest that HCC in cryptogenic cirrhosis may be behaving differently to other types of HCC. In our cohort of patients, more single nodular type of HCC was seen in cryptogenic cirrhosis, and diffuse-type tumors were more common in patients with alcoholic cirrhosis.

Our research findings reveal that NAFLD is the predominant liver disease burden in the country. Unfortunately, with rising diabetes and obesity, the trend is expected to worsen in the future. Nonalcoholic fatty liver disease is a major problem in the community in both adults and children. In the absence of an accepted therapeutic option other than weight reduction and lifestyle modifications, the emphasis should be on preventive strategies initiated from childhood.
